# Supplementary material for: Super-formable pure magnesium at room temperature
Source: Nat Commun. 2017 Oct 17;8:972. doi: 10.1038/s41467-017-01330-9 (PMC5715137; doi:10.1038/s41467-017-01330-9)
Supplement: Supplementary file 1 — Supplementary Information [file 41467_2017_1330_MOESM1_ESM.pdf]

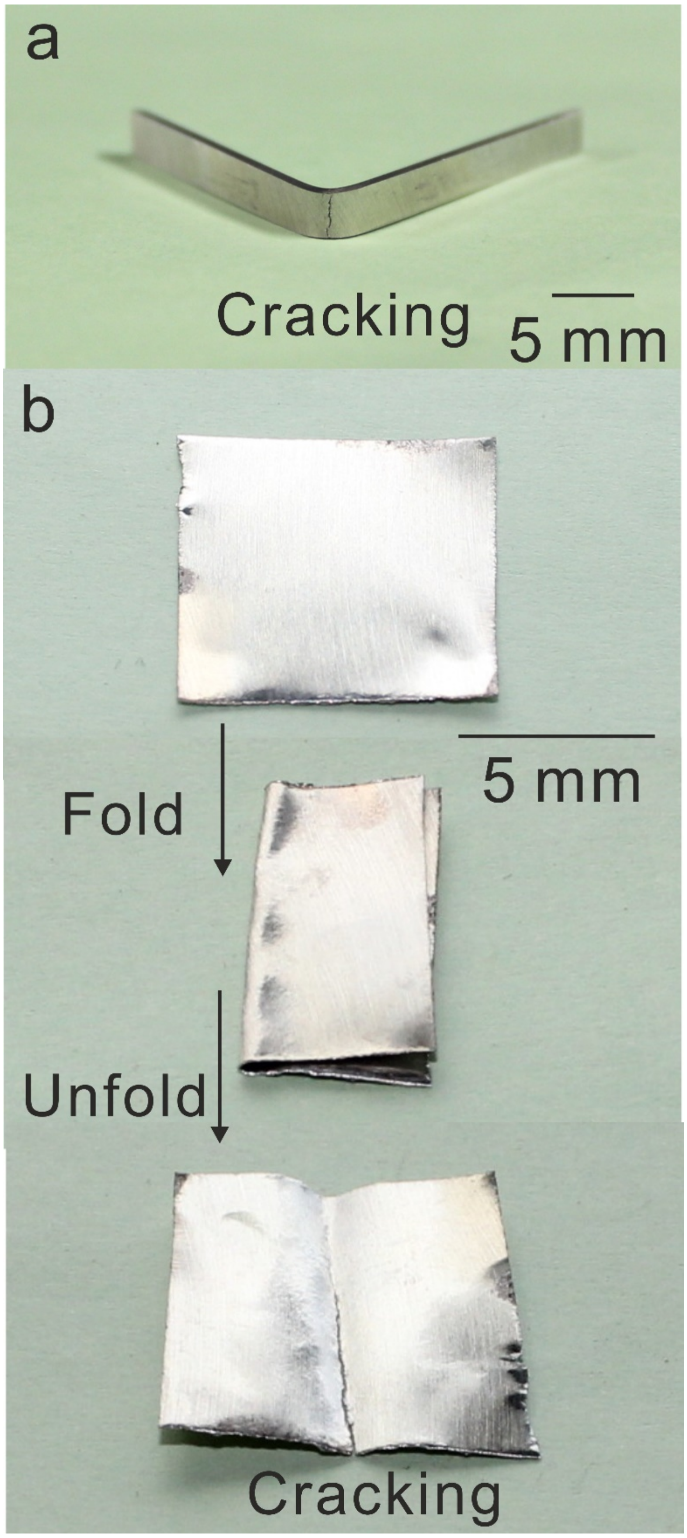

Supplementary Figure 1. (a) Photo showing a 1 mm-thick strip that was hot-rolled at 400°C and annealed at 350°C and bent by ~95° at room temperature and subsequently fractured. (b) Photo showing a hot-rolled and annealed 0.12 mm-thick foil, folded and unfolded, with significant cracking.

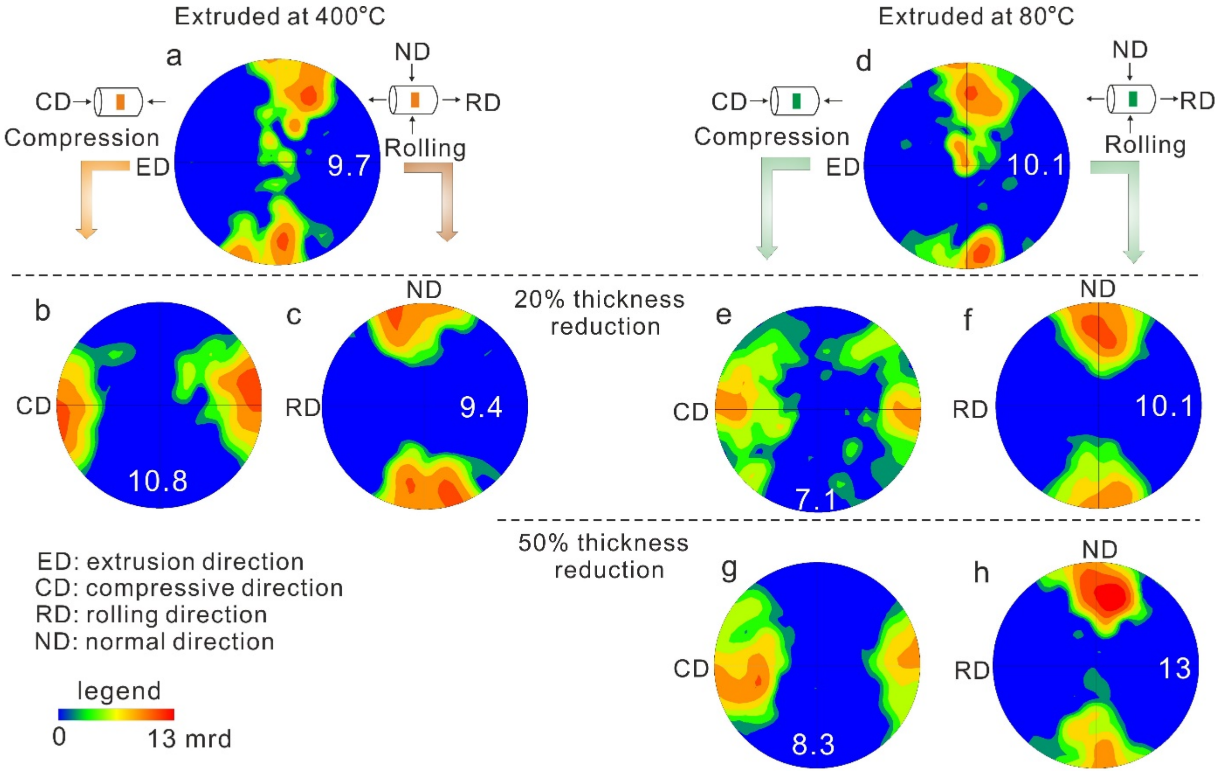

Supplementary Figure 2. (0001) pole figures showing texture of (a–c) 400 °C and (d–h) 80 °C extruded specimens in (a, d) as-extruded state, and (b) compressed and (c) cold rolled by 20% for the 400 °C extruded specimen, and (e, g) compressed and (f, h) cold rolled by 20% and 50% thickness reduction for the 80 °C extruded specimen. The number in each pole figure indicates the maximum texture intensity in multiple of random distribution (mrd).

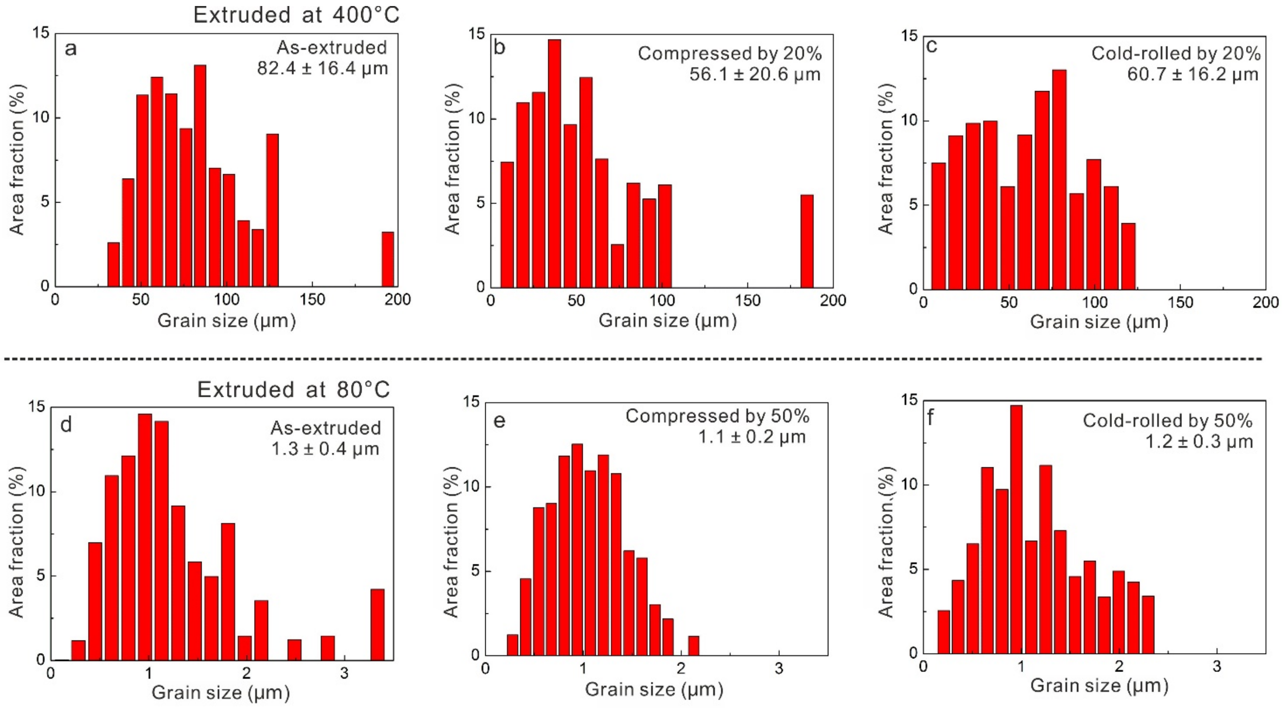

Supplementary Figure 3. Grain size distribution of specimens extruded at (a–c) 400 °C and (d–f) 80 °C. Specimens are in the (a, d) as-extruded state, (b, e) compressed state, and (c, f) cold-rolled state.

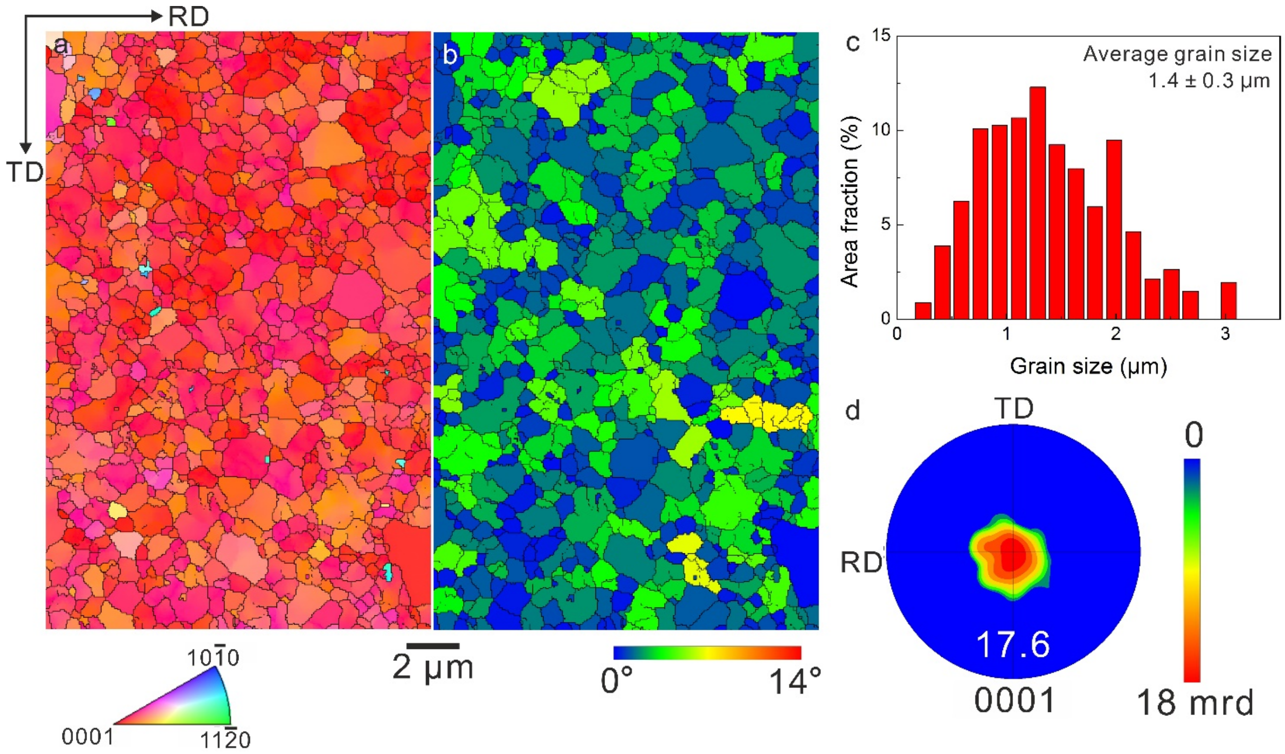

Supplementary Figure 4. TKD maps showing (a) grain orientation and (b) orientation spread inside individual grains in the cold-rolled foil shown in Figure 1c. (c) Grain size distribution, and (d) (0001) pole figure obtained from (a). The number in the pole figure indicates the maximum texture intensity in multiple of random distribution (mrd).

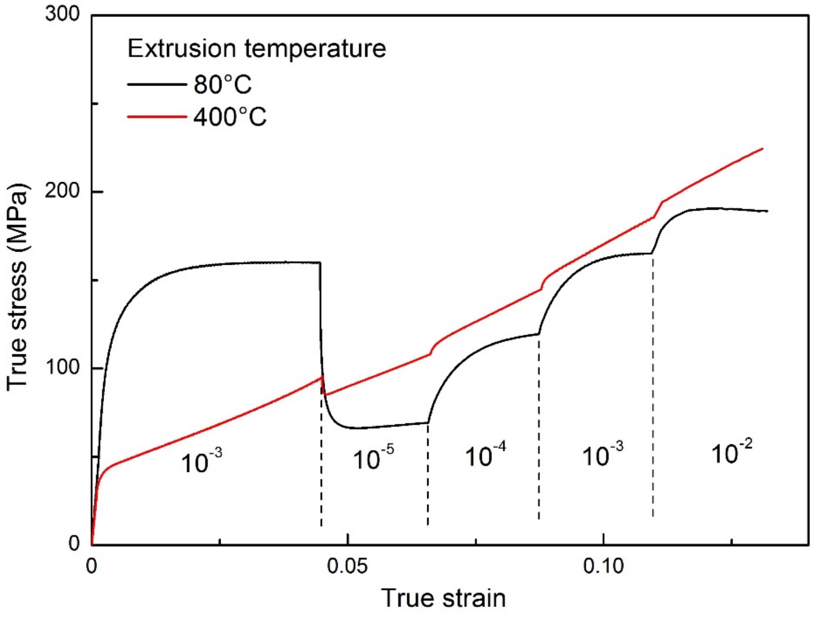

Supplementary Figure 5. Room temperature true stress-strain curves showing strain-rate jump tests of specimens extruded at 80 °C and 400 °C, compressed at different strain rates in the range 10^{-5} – 10^{-2} s^{-1}.

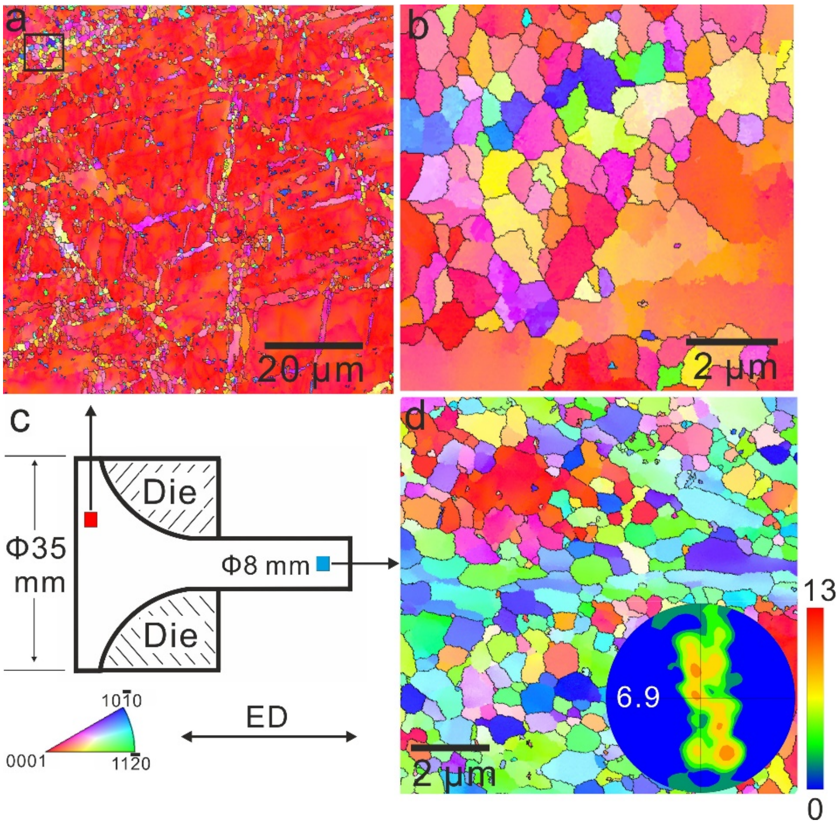

Supplementary Figure 6. (a) Electron backscattered diffraction orientation map showing the microstructure of the residual portion of an originally cylindrical billet of pure Mg that had not passed through the extrusion die (area marked red in (c)), (b) a higher magnification of the area marked by the dark rectangular frame in (a). (c) Schematic diagram showing a specimen partially extruded at room temperature. (d) Transmission Kikuchi diffraction orientation map and corresponding (0001) pole figure showing the microstructure of the specimen, however from the extruded portion marked blue in (c). The number in the pole figure indicates the maximum texture intensity in multiple of random distribution (mrd).

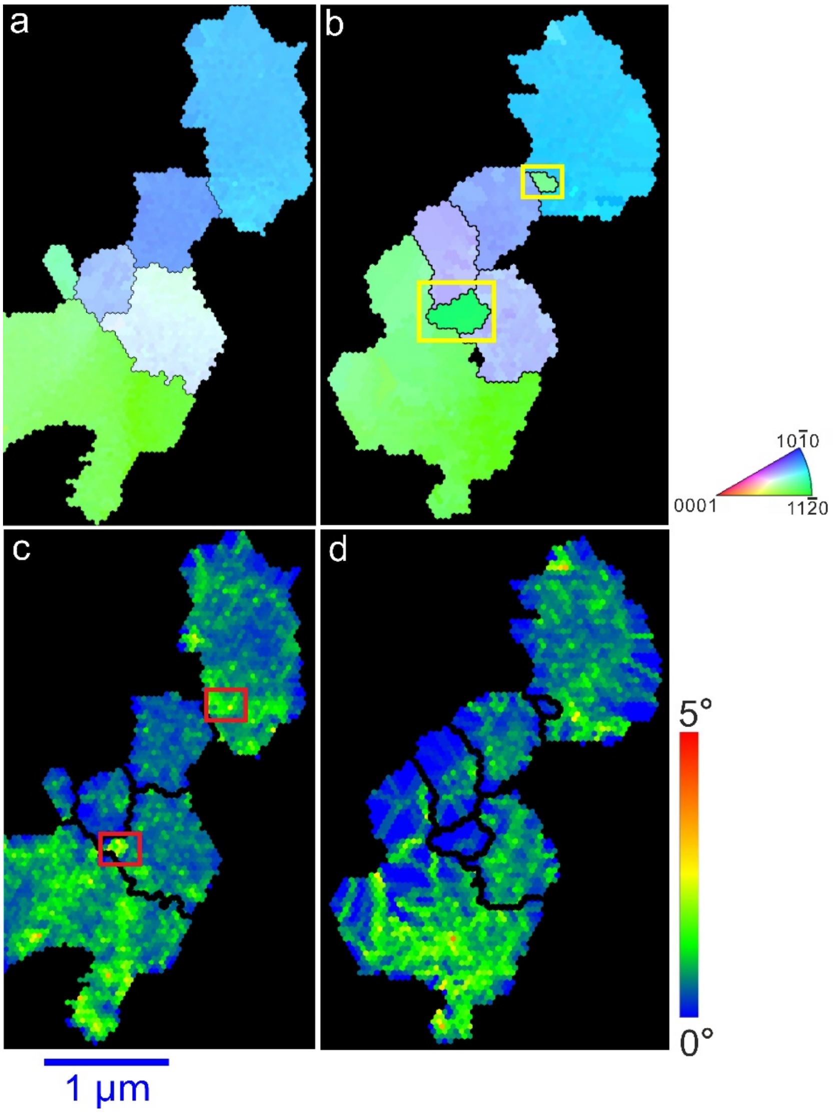

Supplementary Figure 7. *Quasi-in-situ* Electron backscattered diffraction (a, b) orientation and (c, d) kernel average misorientation strain maps showing formation of dynamic recrystallized grains during room temperature compression test of a sample extruded at 80 °C. (a, c) Before and (b, d) after 6% height reduction in the direction perpendicular to the cross-section area. Two essentially strain-free grains, marked by yellow rectangular frames in (b), form in regions with higher strains, highlighted by red rectangular frames in (c).
